# Derivation of Mesenchymal Stromal Cells from Pluripotent Stem Cells through a Neural Crest Lineage using Small Molecule Compounds with Defined Media

**DOI:** 10.1371/journal.pone.0112291

**Published:** 2014-12-02

**Authors:** Makoto Fukuta, Yoshinori Nakai, Kosuke Kirino, Masato Nakagawa, Kazuya Sekiguchi, Sanae Nagata, Yoshihisa Matsumoto, Takuya Yamamoto, Katsutsugu Umeda, Toshio Heike, Naoki Okumura, Noriko Koizumi, Takahiko Sato, Tatsutoshi Nakahata, Megumu Saito, Takanobu Otsuka, Shigeru Kinoshita, Morio Ueno, Makoto Ikeya, Junya Toguchida

**Affiliations:** 1 Department of Tissue Regeneration, Institute for Frontier Medical Sciences, Kyoto University, Kyoto, Japan; 2 Department of Cell Growth and Differentiation, Center for iPS Cell Research and Application, Kyoto University, Kyoto, Japan; 3 Department of Orthopaedic Surgery, Graduate School of Medical Sciences, Nagoya City University, Nagoya, Japan; 4 Department of Ophthalmology, Kyoto Prefectural University of Medicine, Kyoto, Japan; 5 Department of Clinical Application, Center for iPS Cell Research and Application, Kyoto University, Kyoto, Japan; 6 Department of Reprogramming Science, Center for iPS Cell Research and Application, Kyoto University, Kyoto, Japan; 7 Department of Orthopaedic Surgery, Graduate School of Medicine, Kyoto University, Kyoto, Japan; 8 Institute for Integrated Cell-Material Sciences (WPI-iCeMS), Kyoto University, Kyoto, Japan; 9 Department of Pediatrics, Graduate School of Medicine, Kyoto University, Kyoto, Japan; 10 Department of Biomedical Engineering, Faculty of Life and Medical Sciences, Doshisha University, Kyotanabe, Japan; Stem Cell Research Institute, Belgium

## Abstract

Neural crest cells (NCCs) are an embryonic migratory cell population with the ability to differentiate into a wide variety of cell types that contribute to the craniofacial skeleton, cornea, peripheral nervous system, and skin pigmentation. This ability suggests the promising role of NCCs as a source for cell-based therapy. Although several methods have been used to induce human NCCs (hNCCs) from human pluripotent stem cells (hPSCs), such as embryonic stem cells (ESCs) and induced pluripotent stem cells (iPSCs), further modifications are required to improve the robustness, efficacy, and simplicity of these methods. Chemically defined medium (CDM) was used as the basal medium in the induction and maintenance steps. By optimizing the culture conditions, the combination of the GSK3β inhibitor and TGFβ inhibitor with a minimum growth factor (insulin) very efficiently induced hNCCs (70–80%) from hPSCs. The induced hNCCs expressed cranial NCC-related genes and stably proliferated in CDM supplemented with EGF and FGF2 up to at least 10 passages without changes being observed in the major gene expression profiles. Differentiation properties were confirmed for peripheral neurons, glia, melanocytes, and corneal endothelial cells. In addition, cells with differentiation characteristics similar to multipotent mesenchymal stromal cells (MSCs) were induced from hNCCs using CDM specific for human MSCs. Our simple and robust induction protocol using small molecule compounds with defined media enabled the generation of hNCCs as an intermediate material producing terminally differentiated cells for cell-based innovative medicine.

## Introduction

In order to apply human pluripotent stem cells (hPSCs) to innovative medicine, such as cell therapy, disease modeling, and drug discovery, robust and efficient methods to produce the desired cell types without contaminating undesired cells are indispensable [1]. Since the contamination of hPSCs, in particular, may cause serious adverse effects, careful monitoring, which requires a considerable amount of time and cost, has to be conducted. Therefore, it would be beneficial to have intermediate cells between hPSCs and terminally differentiated cells, which are proved to have no contaminated hPSCs, contain limited but multiple differentiation properties, and stably proliferate without phenotypic changes. One of the promising candidates with such features is the neural crest cell (NCC) [2].

The neural crest emerges at the border of the neural and non-neural ectoderm in gastrula embryos during vertebrate development [3]. Cells in the neural crest, and later in the dorsal part of the neural tube, eventually delaminate and migrate throughout the body while retaining their characteristic phenotype [4]. When they reach their target tissues, NCCs differentiate into specific cell types depending on the location [5]. NCCs give rise to the majority of cranial bone, cartilage, smooth muscle, and pigmented cells in the cranial region, as well as neurons and glia in the peripheral nervous system [3]–[5]. Cardiac NCCs are known to contribute to valves in the heart, while vagal NCCs differentiate into enteric ganglia in the gut [6]. NCCs give rise to neurons and glia in the peripheral nervous system in the trunk region, secretory cells in the endocrine system, and pigmented cells in the skin.

Using a lineage-tracing system, rodent neural crest-derived cells were detected in adult tissues such as bone marrow, and still retained multipotent differentiation properties, which indicated that these cells are one of the cell-of-origin of multipotent mesenchymal stromal cells (MSCs) [7], [8]. Therefore, the production of human MSCs (hMSCs) from hPSCs via NCC lineage is a promising approach for the use of hPSCs in innovative medicine [9], [10]. A considerable number of studies have been dedicated to establishing robust and efficient induction methods from hPSCs to hNCCs in the past decade [11]–[13]. However, most of these studies used non-human stromal feeder cells or only achieved low induction efficiencies. An ideal method from the standpoint of clinical applications is free from xeno-materials, such as feeder cells or serum, and can be performed using a chemically defined medium (CDM). Two groups have published protocols that are compatible with these requirements [14],[15]. The first group employed a two-step approach, in which hPSCs were firstly dissociated into single cells and cultured with CDM for two weeks for the adaptation. Cells were then cultured with CDM that was supplemented with an activator of Wnt signaling and inhibitor of Activin/Nodal/TGFβ signaling, but was free from BMP signaling modulation [14]. The other group used MEF-conditioned hESC media for the initial step, and replaced it with knockout serum replacement (KSR)-based medium, which thereafter was gradually replaced with an increasing amount of N2 media. They employed an inhibitor for BMP signaling in addition to an inhibitor for Activin/Nodal/TGFβ signaling during the initial 3 days, and then replaced them with an activator of Wnt signaling [15]. Therefore, the requirement for signal modulators, particularly BMP signaling inhibitors, remains controversial. Further modifications are still needed to improve the robustness, efficacy, and simplicity of these methods.

We here developed a robust and efficient induction protocol using CDM containing inhibitors for TGFβ signaling and GSK3β, but not for BMP signaling with minimal growth factors. The protocol very efficiently induced hNCCs (70–80%) from hPSCs irrespective of the type (hESCs vs hiPSCs) or generating method (viral-integrated vs plasmid-episomal). Genome-wide analyses revealed that induced hNCCs retained their gene expression profile as NCCs even after 10 passages. As for differentiation properties, induced hNCCs successfully differentiated into peripheral neurons, glia, melanocytes and corneal endothelial cells. In addition, induced hNCCs were able to produce cells comparable to hMSCs, which were free from contaminated hPSCs and could differentiate into osteo-, chondro-, and adipogenic cells. Furthermore, using iPSCs generated and maintained under feeder-free and xeno-free culture systems, we successfully induced hNCCs, hMSCs, and osteogenic cells using chemically defined media.

## Materials and Methods

### Ethics statement

The experimental protocols dealing human subjects were approved by the Ethics Committee of the Department of Medicine and Graduate School of Medicine, Kyoto University. Written informed consent was provided by each donor.

### Cell lines

hESCs (H9, KhES1, and KhES3) and hiPSCs (414C2 and 201B7) were used in this study [16]–[19] They were maintained on SNL feeder cells [20] in Primate ES cell medium (ReproCELL, Tokyo, Japan) supplemented with 4 ng/ml recombinant human FGF2 (WAKO, Osaka, Japan). 987A3, hiPSCs generated and maintained under feeder-free and xeno-free culture systems from human primary fibroblasts, were maintained on iMatrix-551 (rLN511E) (Nippi, Tokyo, Japan)-coated cell culture plates with StemFit (Ajinomoto, Tokyo, Japan) as described previously [21]. Bone marrow derived hMSCs were obtained from donors and used in our previous study [22]. Human corneal endothelial cells were isolated from human corneal tissues obtained for research purpose from SightLife (Seattle, WA, USA).

### Culture media and reagents

mTeSR1 medium (STEMCELL Technology, Vancouver, Canada) was used for the feeder-free culture of PSCs. The induction and maintenance of hNCCs were performed using previously reported CDM [23], which contains Iscove's modified Dulbecco's medium/Ham's F-12 1∶1, 1x chemically defined lipid concentrate (GIBCO, Grand Island, NY, USA), 15 µg/ml apo-transferrin (Sigma, St. Louis, MO, USA), 450 µM monothioglycerol (Sigma), 5 mg/ml purified BSA (99% purified by crystallization; Sigma), 7 µg/ml Insulin (WAKO), and penicillin/streptomycin (Invitrogen, Carlsbad, CA, USA). Culture dishes were coated with growth factor-reduced Matrigel (BD, Bedford, MA, USA) or fibronectin (Millipore, Bedford, CA, USA). EGF (R&D, Minneapolis, USA) and FGF2 were used to maintain hNCCs [24]. SB431542 (SB) (Sigma), CHIR99021 (CHIR) (WAKO), BMP4 (R&D), DMH1 (Tocris, Bristol, UK), LDN193189 (Stemgent, Cambridge, MA, USA), and recombinant human Noggin (R&D) were used to modulate growth factor signals. Retinoic acid (RA) (Sigma) was used to modulate hNCCs.

### Fluorescence-Activated Cell Sorting (FACS)

FACS was performed by AriaII (BD) according to the manufacturer's protocol. The antibodies used in FACS were listed in Table S1. In all experiments, FACS histograms of isotype controls were similar to those without antibodies; therefore, histograms without antibodies were used as control populations.

### Immunocyto- and immunohistochemistry

Prior to performing immunostaining with antibodies, cells on plates were fixed with 4% paraformaldehyde at 4°C for 15 minutes, washed two times with PBS, and incubated with 0.3% TritonX100 at 4°C for 30 minutes as the surface-active agent for penetration processing, and any nonspecific binding was blocked with 2% skim milk/PBS at 4°C for 1 hour. Cornea samples obtained from rabbits euthanized three days after the injection of cells were fixed with 4% paraformaldehyde and incubated in 1% bovine serum albumin (BSA) (Sigma) to block other bindings. DAPI (1∶5000; Sigma) was used to counterstain nuclei. The primary antibodies used in this study were summarized in Table S1. The observation and assessment of samples were performed with BZ-9000E (Keyence, Osaka, Japan).

### RT-PCR and qPCR

Total RNA was purified with the RNeasy Mini kit (Qiagen, Valencia, CA, USA) and treated with the DNase-one kit (Qiagen) to remove genomic DNA. One microgram of total RNA was reverse transcribed for single-stranded cDNA using a random primer and Superscript III reverse transcriptase (Invitrogen), according to the manufacturer's instructions. PCR was performed with ExTaq (Takara, Shiga, Japan). Quantitative PCR with the Thunderbird SYBR qPCR Mix (TOYOBO, Osaka, Japan) was performed using the StepOne real-time PCR system (Applied Biosystems, Forester City, CA, USA) in duplicate or triplicate. Primer sequences were listed in Table S2.

### cDNA microarray

Total RNA was prepared using the RNeasy Mini Kit (Qiagen). cDNA was synthesized using the GeneChip WT (Whole Transcript) Sense Target Labeling and Control Reagents kit as described by the manufacturer (Affymetrix, Santa Clara, CA, USA). Hybridization to the GeneChip Human Gene 1.0 ST expression arrays, washing, and scanning were performed according to the manufacturer's protocol (Affymetrix). Expression values were calculated using the RMA summarization method and the data obtained were analyzed by GeneSpring GX 11.5.6 (Agilent Technologies, Santa Clara, CA, USA) for correlation coefficients, scatter plots, a volcano plot, heat maps, and hierarchical clustering (Distance metrics: Pearson's Centered, Linkage rule: Average). Differentially expressed genes were identified by statistical analyses and fold changes. Statistical analyses were performed using a one-way ANOVA with a Benjamini and Hochberg False Discovery Rate (BH-FDR  = 0.01) multiple testing correction followed by Tukey HSD post hoc tests (GeneSpring GX). Microarray data have been submitted to the Gene Expression Omnibus (GEO) public database at NCBI, and the accession number is GSE 60313. Data for hBM90, 91, and 94 have already been described [22]. Data from GSE44727 and GSE45223 were used for a comparison analysis in Figure S3.

### Differentiation of hPSC-derived hNCCs

#### Peripheral neuronal differentiation

Sorted hNCCs were cultured in CDM supplemented with 10 µM SB and 1 µM CHIR as a sphere using the hanging drop technique (1×10^4^ cells per sphere) as previously described [25]. Twenty-four hours after the hanging drop culture, spheres were plated onto Polyornithine/laminin/fibronectin (PO/Lam/FN)-coated plates in DMEM/F12 (Invitrogen) supplemented with 1 x N2 supplement (GIBCO), 1 x GlutaMAX (Invitrogen), 20 ng/ml FGF2, and 20 ng/ml EGF. These cells were cultured for two days under these conditions and the medium was then replaced with DMEM/F12 supplemented with 1 x N2 supplement, 1 x GlutaMAX, and 10 ng/ml BDNF (R&D), GDNF (R&D), NT-3 (R&D), and NGF (R&D). The medium was changed every 3 days and passages were performed every week [26]. Differentiation was confirmed by immunostaining for peripherin, Tuj-1, and GFAP 3 weeks after induction.

#### Melanocyte differentiation

Cells were plated onto fibronectin-coated dishes in CDM supplemented with 10 µM SB and 1 µM CHIR. Melanocyte induction was performed the next day with CDM supplemented with 1 µM CHIR, 25 ng/ml BMP4, and 100 nM endothelin-3 (American Peptide Company, Sunnyvale, CA, USA) [15], [27], [28]. The medium was changed every other day. Differentiation was confirmed by induction of the *MITF* and *c-KIT* genes on day 7.

#### Corneal endothelial cell differentiation

Cells were induced to corneal endothelial cells with corneal endothelial cell-conditioned CDM. Conditioned CDM was derived by collecting medium from cultured human corneal endothelial cells [29]. The selective ROCK inhibitor Y-27632 (WAKO) was used on the first day of the induction. The medium was changed every two days, and cells were analyzed by immunocytochemistry after twelve days. RT-qPCR was performed 3, 5, and 8 days after the induction.

### Induction of hMSCs from hNCCs

Cells were plated onto tissue culture dishes (BD) at a density of 6.5×10^4^ cell/cm^2^ in CDM supplemented with 10 µM SB and 1 µM CHIR. The medium was replaced the next day with αMEM (Nacalai Tesque, Tokyo, Japan) supplemented with 10% fetal bovine serum (FBS) (Nichirei Inc., Tokyo, Japan) [14], [26]. The morphology of cells started to change approximately 4 days after the induction. Passages were performed every week using 0.25% trypsin-EDTA (GIBCO) at a density of 1×10^4^ cells/cm^2^. hMSC markers (CD73, CD44, CD45 and CD105) were analyzed by FACS 14 days after the hMSC induction. We used STK2 (DS Pharma Biomedical, Osaka, Japan) as the MSC medium and tissue culture dishes coated with fibronectin for the hMSC induction under chemically defined media conditions.

### Differentiation of hNCC-derived hMSCs

#### Osteogenic differentiation

A total of 2.5×10^5^ induced hMSCs/well were seeded on 6-well dishes (BD) and cultured in osteogenic induction medium, αMEM, 10% FBS, 0.1 µM dexamethasone, 50 µg/ml ascorbic acid, and 10 mM β-glycerophosphate for 2 weeks for osteogenic differentiation [24]. STK3 (DS Pharma) was used as the osteogenic induction medium instead of the osteogenic induction medium to achieve osteogenic differentiation under chemically defined media conditions. The culture medium was changed every other day for 1 week. Differentiation properties were confirmed by the formation of calcified nodules, as detected with Alizarin Red staining. Briefly, culture wells were washed twice in phosphate-buffered saline (PBS) and fixed for 10 minutes at room temperature in 100% ethyl alcohol. The Alizarin Red solution (40 mM, pH 4.2) was applied to the fixed wells for 10 min at room temperature. Non-specific staining was removed by several washes with water.

#### Chondrogenic differentiation

Two-dimensional chondrogenic induction was performed as previously described [30]. Briefly, cells (1.5×10^5^) that induced hMSCs were suspended in 5 µl of chondrogenic medium (DMEM: F12 (Invitrogen), 1% (v/v) ITS1 mix (BD), 0.17 mM AA2P, 0.35 mM Proline (Sigma), 0.1 mM dexamethasone (Sigma), 0.15% (v/v) glucose (Sigma), 1 mM Na-pyruvate (Invitrogen), 2 mM GlutaMax, and 0.05 mM MTG supplemented with 40 ng/ml PDGF-BB and 1% (v/v) FBS (Nichirei)), and were subsequently transferred to fibronectin-coated 24-well plates (BD). A total of 1 ml of the chondrogenic medium was added after 1 hour. TGFβ3 (R&D) was subsequently added at 10 ng/ml on days 3 to 6, and BMP4 was added to a concentration of 50 ng/ml on day 10. Micromass cultures were maintained at 37°C under 5% CO_2_ and 5% O_2_ for 16 days. Differentiation properties were confirmed by Alcian Blue staining. Briefly, induced cells were fixed for 30 minutes with 10% formalin (Sigma) and rinsed with PBS. These cells were then stained overnight with Alcian Blue solution (1% Alcian Blue (MUTO PURE CHEMICAL CO., LTD, Tokyo, Japan) in 3% glacial acetic and 1% HCl, pH 1) and destained with the acetic acid solution.

#### Adipogenic differentiation

Cells were seeded onto 6-well tissue culture dishes at a density of 5.0×10^5^ cells/well for adipogenic differentiation, and were cultured in αMEM containing 10% FBS, 1 mM dexamethasone, 10 mg/ml insulin, and 0.5 mM isobutylxanthine for 3 weeks [31]. Induced cells were fixed in 10% formalin for 1 hour at room temperature, followed by 20 minutes in 0.3% Oil Red O staining solution (Sigma).

## Results

### Derivation of p75^high^ cells from hPSCs

**Figure 1:**
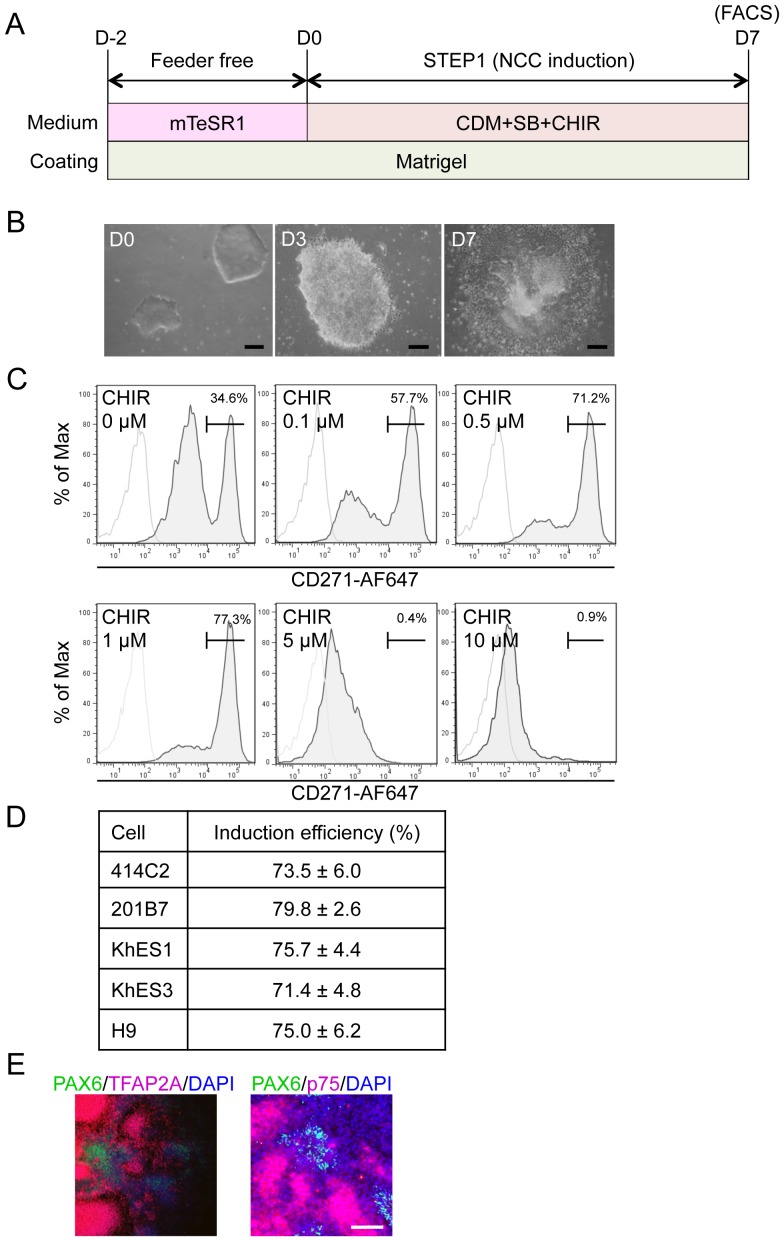
Induction of p75^high^ cells from hPSCs. A) Schematic representation of the protocol. B) Morphology of colonies during the induction. Phase contrast images were taken on days 0, 3, and 7. Scale bar, 200 µm. C) The fraction of p75-positive cells in 201B7 cells was treated with SB431542 (SB) (10 µM) and CHIR99021 (CHIR) (indicated concentration) for seven days, stained with an anti-p75 antibody, and analyzed by FACS. D) Fraction of the p75^high^ population induced by SB (10 µM) and CHIR (1 µM) from hESCs (KhES1, KhES3, H9) and hiPSCs (414C2, 201B7). Average ± SD. N = 3, biological triplicate. E) Immunocytochemical analyses of colonies on day 7 (201B7). Cells were stained with antibodies against PAX6, TFAP2A, and p75. Scale bar, 100 µm.

To transfer hPSCs from feeder to feeder-free culture conditions, colonies were dissociated into small cell clumps (about 10 cells/clumps) by pipetting several times, seeded on matrigel-coated dishes (2–4 clumps/cm^2^), and cultured with mTeSR1 medium for two days. hNCC induction was then initiated by substituting CDM supplemented with chemicals (Figure 1A). Cells gradually migrated from colonies and proliferated during the induction (Figure 1B). Cells were harvested after 7 days of being induced, and were subsequently sorted according to the expression of p75 (Figure 1C). We detected two peaks in p75-positive populations, designated p75^low^ and p75^high^, and the efficiency of hNCC induction was evaluated based on the fraction of p75^high^ cells.

The effects of SB, which has been shown to inhibit Activin/Nodal/TGFβ signaling and induce neural cells and hNCCs from hPSCs without the help of other chemicals, were firstly evaluated [14]. In accordance with the reported data, CDM supplemented with SB successfully delivered p75^high^ cells with a PAX6-positive neuroectoderm from 201B7 (date not shown), while the induction efficiency of p75^high^ cells was approximately 35% (0 µM in Figure 1C). The activation of Wnt signaling was previously shown to play a key role in the induction of hNCCs [14], [15], and can be achieved using the GSK3β inhibitor BIO or CHIR. Therefore, we attempted to determine the most effective concentration of CHIR with a fixed concentration of SB (10 µM) to induce p75^high^ cells. The results obtained revealed that CHIR successfully induced p75^high^ cells in a dose-dependent manner up to 1 µM, whereas higher concentrations of CHIR markedly inhibited the production of p75^high^ cells (Figure 1C). We finally examined the effects of BMP signaling on this induction. The addition of BMP4 markedly inhibited the production of p75^high^ cells, and these results were compatible with BMP signal inhibiting neural differentiation (Figure S1A). However, the treatment with DMH1 (10 µM), a specific inhibitor of SMAD1/5/8 phosphorylation, also reduced the p75^high^ fraction (Figure S1B). The inhibitory effect of DMH1 on the induction of p75 was confirmed at different dosages, and other cytoplasmic (LDN193189) or extracellular (Noggin) inhibitors for BMP signaling also decreased the efficiency (Figure S1C). Therefore, the combination of SB (10 µM) and CHIR (1 µM) most effectively induced p75^high^ cells from 201B7 hiPSCs. This result was reproduced in other hPSCs such as hESCs (H9, KhES1, and KhES3) and episomal hiPSCs (414C2) (Figure 1D). Most cells outgrowing from colonies were stained with NCC markers, p75 and TFAP2A, whereas the cells in colonies were positive for PAX6, a marker for the neuroectoderm (Figure 1E).

### p75^high^ cells expressed early NCC markers

**Figure 2:**
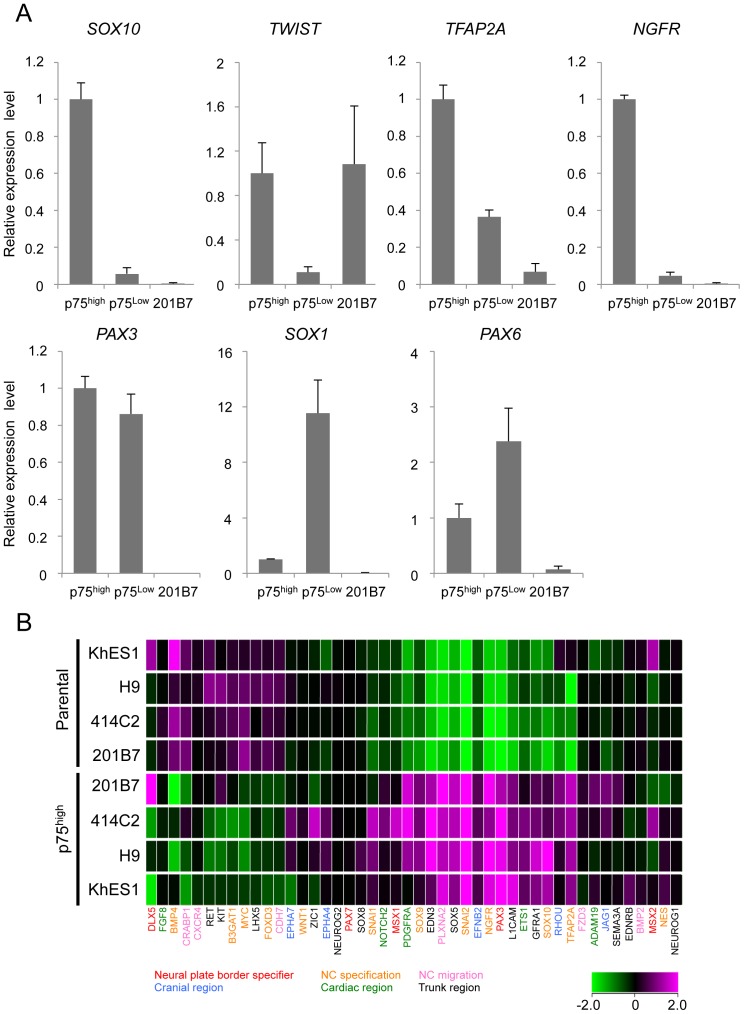
Expression profiles of sorted p75^high^ cells. A) The expression of marker genes in sorted p75^high^ and p75^low^ cells. The mRNA expression of each gene was analyzed by RT-qPCR in undifferentiated 201B7 (hiPSCs) and sorted p75^low^ and p75^high^ cells, and was shown as a relative value using the level in sorted p75^high^ cells as 1.0. Average ± SD. N = 3, biological triplicates. B) Clustering analyses of NCC markers in p75^high^ populations from several hESC and hiPSC lines. Marker genes for each sub-population of NCCs were labeled using the indicated colors.

The expression of marker genes were compared between p75^high^ and p75^low^ cells (Figure 2A). Sorted p75^high^ cells expressed a number of genes in the early stage of NCCs, such as *SOX10*, *TWIST*, and *TFAP2A* genes. In contrast, the expression of these genes was significantly lower in the p75^low^ fraction than in p75^high^ fraction. The expression of *PAX3*, which is a marker both for NCCs and neurons, was high in both the p75^high^ and p75^low^ fractions. The expression of *PAX6* and *SOX1*, which are neural markers, was higher in the p75^low^ fraction, which is consistent with some populations of p75-negative or TFAP2A-negative cells expressing *PAX6* (Figure 1E). These results indicated the relative enrichment of NCC cells in the p75^high^ cell population.

In an attempt to further characterize p75^high^ cells, genome-wide expression profiles were compared between sorted p75^high^ cells and their corresponding hPSCs using a cDNA microarray (Affymetrix Gene 1.0 ST), and we found that the overall profiles of p75^high^ cells derived from several PSCs were similar to each other (Figure S2). Based on the previous report [32], 46 genes were selected as markers for distinct NC-subpopulations and the expression level of these genes were compared between hNCCs and parental PSCs (Figure 2B). hNCCs in this study highly expressed early stage-related genes such as neural plate border specifier (PAX3) and NC specification (SNAI2, NGFR, TFAP2A, SOX9, and SOX10), but also some region-specifying genes (EFNB2 for cranial, PDGFRA for cardiac, and SOX5 for trunk region), suggesting the heterogeneous population of p75^high^ cells, which were designated hNCCs hereafter. The profiles of the current hNCCs were compared with those of two PSC-derived NCCs, which were induced by different protocols in previous studies [15], [32] (Figure S3). Although the three types of PSC-derived NCCs all highly expressed some genes, such as SNAI2, their expression profiles were considerably different, suggesting the protocol-dependent heterogeneity of PSC-derived NCCs.

### Sustained expansion of hNCCs with original characteristics

**Figure 3:**
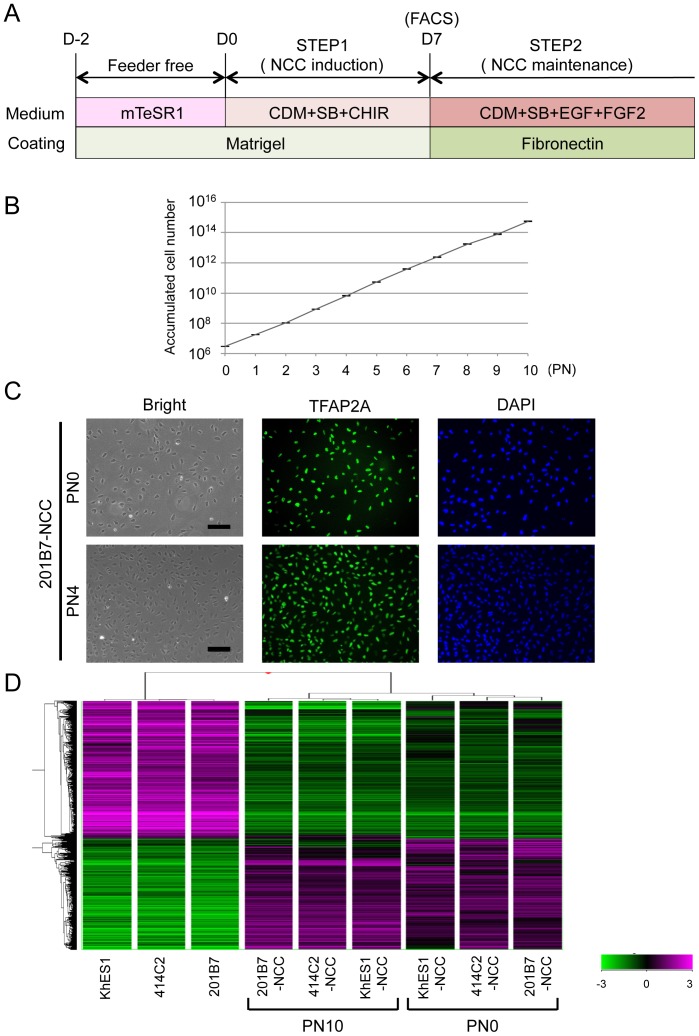
Sustained expansion of hNCCs with original characteristics. A) Schematic representation of the culture conditions. B) Growth profile of 201B7-derived hNCCs. Average ± SD. N = 3, biological triplicate. C) Phase contrast images and immunostaining of TFAP2A in 201B7-derived hNCCs at PN0 and PN4, Scale bar, 200 µm. D) Hierarchical clustering analyses of hPSCs and hPSC-derived hNCCs at PN0 and PN10.

We investigated whether hNCCs could be stably expanded. The growth of hNCCs cultured in the hNCC induction medium (CDM with SB and CHIR) was very slow (data not shown). We employed a cultured condition using CDM supplemented with SB, EGF (20 ng/ml), and FGF2 (20 ng/ml) based on the findings of previous studies [33], and consequently observed marked improvements in growth and the stable proliferation of hNCCs even after 10 passages (Figures 3A, B). The expanded hNCCs maintained their original cell morphology and all cells expressing NCC markers, such as TFAP2A (Figure 3C). The global gene expression profiles of hNCCs after prolonged expansion (PN10) were similar to those of early-passage cells (PN0) (Figure S4A and S4B, correlation coefficient  = 0.96 to 0.98) and markedly different from those of original hPSCs (Figures 3D and S4C).

### Modulation of the characteristics of hNCCs by insulin and retinoic acid (RA)

**Figure 4:**
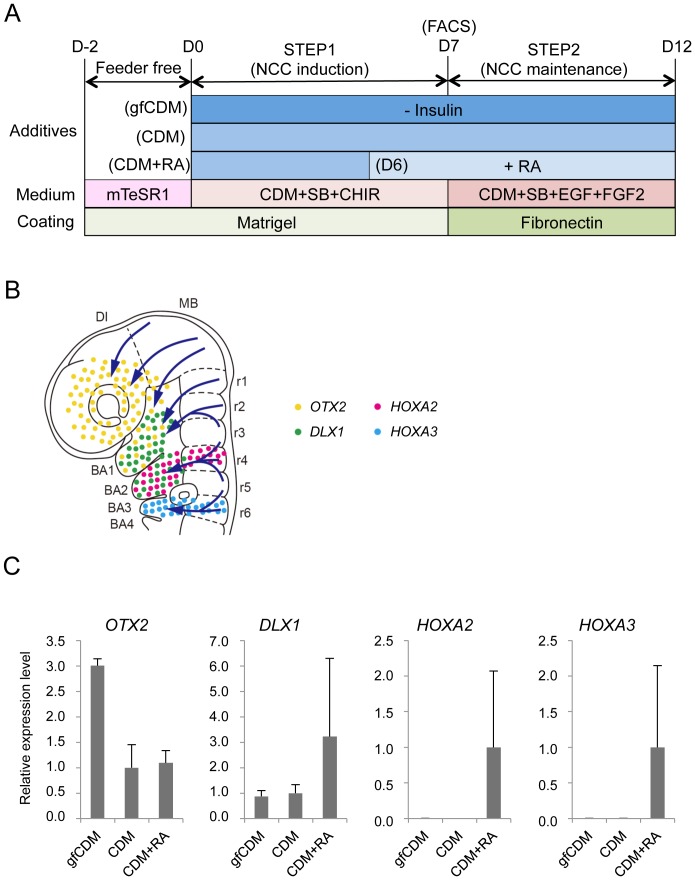
Modulation of regional characteristics of hNCCs. A) Schematic representation of culture conditions for the induction and maintenance of hNCCs. RA, retinoic acid (100 nM). B) Schematic distribution of marker-positive cells in the murine embryo. DI, diencephalon; MB, midbrain; BA1 to BA4, branchial arch 1 to branchial arch 4; r1 to r6; rhombomere 1 to rhombomere 6. C) The mRNA expression of regional specifier genes in hNCCs. p75^high^ cells were collected at the end of the hNCC induction by FACS and seeded onto fibronectin-coated dishes. RNAs were extracted when cells reached a semi-confluent state and used for RT-qPCR. The relative expression level of each gene was demonstrated using the value of cells cultured in CDM (*OTX2* and *DLX1*) or CDM + RA (*HOXA2* and *HOXA3*) as 1.0. Average ± SD. N = 3, biological triplicate.

The results of the microarray analyses revealed that induced hNCCs expressed some genes characteristic to cranial NCCs (high for *OTX2* and *DLX1*; low for *HOXA2* and *HOXA3*) (date not shown). A previous study demonstrated that the depletion of insulin from CDM (growth-factor free CDM; hereafter referred to as gfCDM) induced a more anterior neuroectoderm (rostral hypothalamic progenitor-like cells), while retinoic acid (RA) exhibited posteriorizing activity [15]. Therefore, we compared the expression of regional markers in hNCCs cultured with gfCDM, CDM, and CDM with RA (100 nM) (Figure 4A). As expected, the expression of *OTX2*, a marker for mesencephalic NCCs (Figure 4B) [34], was slightly higher under the gfCDM condition than under the CDM condition (Figure 4C). The *DLX1* gene, a marker for first and second branchial arch NCCs (Figure 4B) [35], was expressed in cells cultured under all conditions, and was the highest in CDM with the RA condition (Figure 4C). The expression of the *HOXA2* and *HOXA3* genes, which are markers of the second and third branchial arches, was negligible under the gfCDM and CDM conditions (Figures 4B, C) [36], [37]. Taken together, these results indicated that the regional identities of hNCCs could be modulated by exogenous signals including insulin and RA.

### Derivation of peripheral neurons, glia, and melanocytes from hNCCs

**Figure 5:**
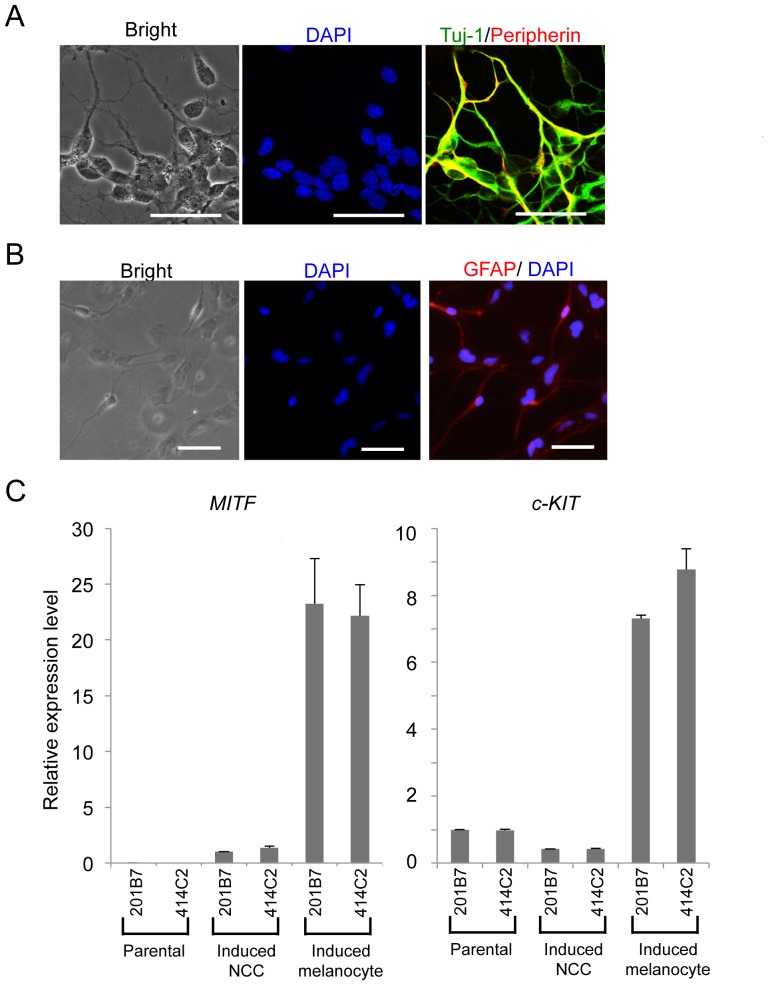
Derivation of peripheral neural cells, glia, and melanocytes from hNCCs. A) Neuronal differentiation of 201B7-derived hNCCs. Cells were stained with an antibody against peripherin (red) and Tuj-1 (green). B) The glial differentiation of 201B7-derived hNCCs. Cells were stained with an antibody against GFAP. Scale bar, 50 µm. C) Melanocyte differentiation of 201B7-derived hNCCs. The mRNA expression levels of *MITF* and *c-KIT* genes were shown as a relative value using the value in 201B7-derived hNCCs and 201B7 as 1.0, respectively. Average ± SD. N = 3, biological triplicates.

We next examined the differentiation potentials of induced hNCCs. Neuronal differentiation was initiated by sphere formation and promoted by culture media containing a mixture of factors (BDNF, GDNF, NGF, and NT-3). Cells expressed β-tubulin and peripherin after 14 days, which indicated differentiation into peripheral neurons (Figure 5A). Further cultivation under the same conditions (4 to 6 weeks) promoted the glial differentiation of hNCCs (Figure 5B).

Melanocytes are well-known derivatives of NCCs. Using a previously described method that included CHIR, EDN3, and BMP4 [15], [38], induced hNCCs expressed microphthalmia-associated transcription factor (*MITF*) and *c-KIT*, markers for melanocytes (Figure 5C). These differentiation properties were compatible with those of NCCs *in vivo*.

### Derivation of corneal endothelial cells from hNCCs

**Figure 6:**
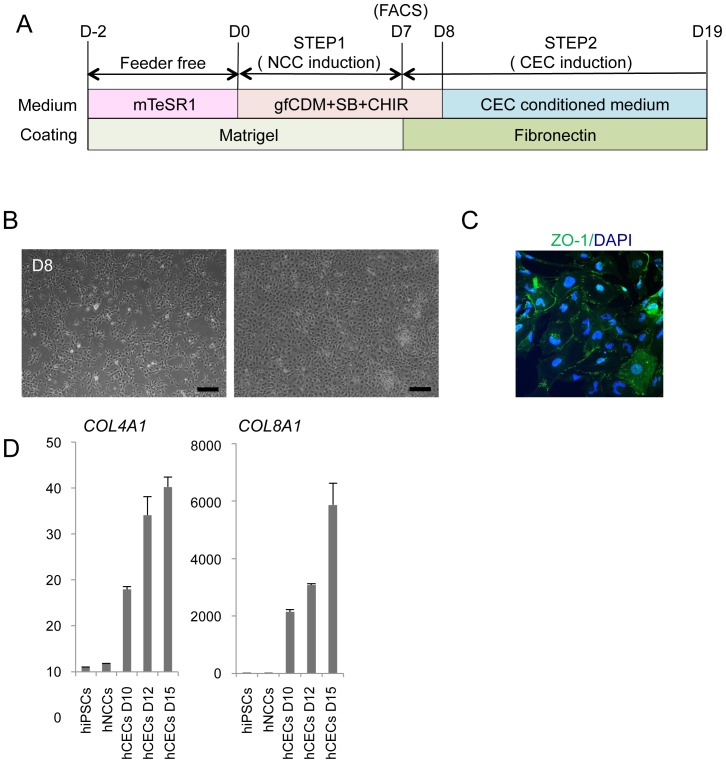
Derivation of corneal endothelial cells from hNCCs. A) Schematic protocol for the induction of corneal endothelial cells. B) Phase contrast images of cells before (D8) and after (D19) the induction. Scale bar, 200 µm. C) The expression of ZO-1 in cells at D12. Cells were stained with an antibody against ZO-1. D) The mRNA expression of corneal endothelial cell marker genes. RNAs were extracted from cells at D10, D12, and D15. The expression level of each gene was demonstrated as a relative value using the value in human primary corneal endothelial cells as 1.0. Average ± SD. N = 3, technical triplicate. We performed this CEC induction twice and confirmed its reproducibility.

Cranial NCCs have been shown to exhibit the ability to differentiate into corneal endothelial cells *in vivo* [39], [40]. Therefore, we examined whether hNCCs grown in gfCDM, which preferentially expressed more anterior NCC markers (Figures 4B, C), could differentiate into cells harboring the characteristics of corneal endothelial cells. When 201B7-derived hNCCs were cultured in the conditioned medium of corneal endothelial cells for twelve days (Figure 6A), cells changed their morphology into that of polygonal corneal endothelial-like cells (Figure 6B) and started to express the corneal endothelial marker, ZO-1 (Figure 6C). Descemet's membrane is known to consist of collagen type 4 and collagen type 8, which are derived from the corneal endothelium [41]. The mRNA expression of the *COL4A1* and *COL8A1* genes was confirmed in induced endothelial-like cells (Figure 6D). These results strongly suggested that the hNCCs induced in this study possessed the characteristics of cranial NCCs, which exhibit the potential to differentiate into cranial NCC-derived structures.

### Derivation of hMSCs from hNCCs

**Figure 7:**
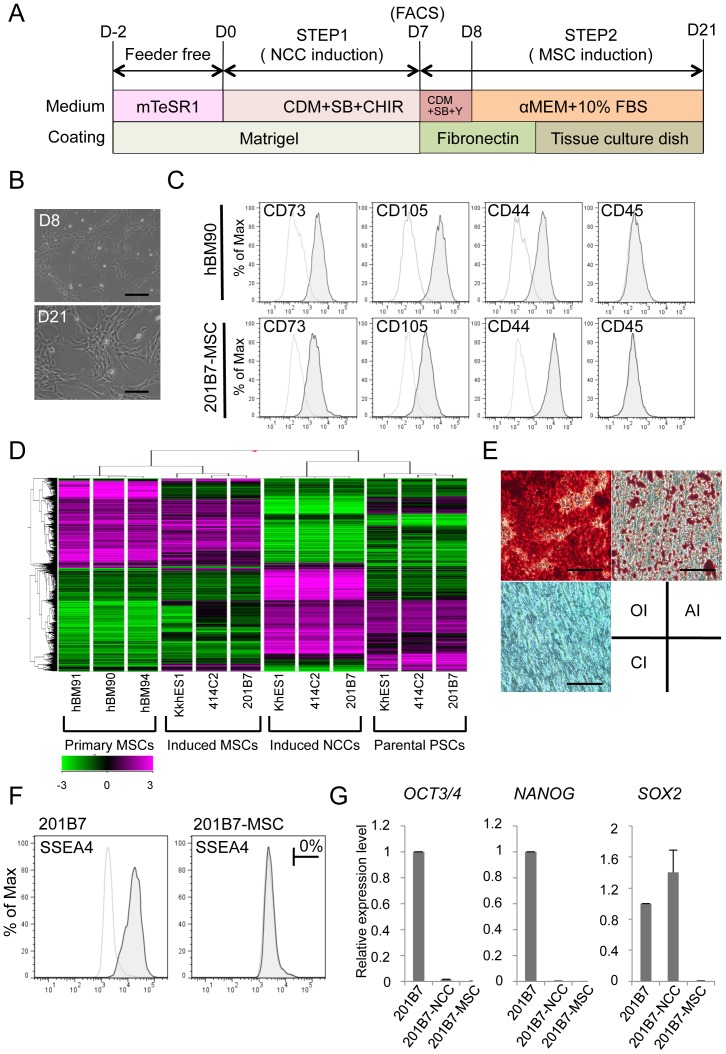
Derivation of hMSCs from hNCCs. A) Schematic protocol for the induction of hMSCs. B) Phase contrast images of cells before (D8) and after (D21) the induction. Scale bar, 200 µm. C) Expression of surface markers in hBM-MSCs (hBM90) and 201B7-derived MSCs (201B7-MSC). D) Hierarchical clustering analyses by genome-wide gene expression profiles. RNAs were extracted from hBM-MSCs (BM90, BM91 and BM94), induced-MSCs, and the corresponding hNCCs and hiPSCs. E) Differentiation properties of induced-MSCs. The induction for osteogenic (OI), chondrogenic (CI), and adipogenic (AI) lineages was performed as described in the Materials and Methods section and evaluated by Alizarin Red staining (OI), Alcian Blue staining (CI), and Oil Red O staining (AI), respectively. Scale bar, 100 µm. F) Population of SSEA4-positive cells. G) The expression levels of pluripotent markers (*OCT3/4*, *NANOG* and *SOX2*) in hPSCs, hNCCs, and hMSCs. Average ± SD. N = 3, biological triplicates.

Cranial NCCs also have differentiation properties toward mesenchymal cells, which construct the cranio-facial skeleton, and may be referred as MSCs [3]. In order to derivate hMSCs from hNCCs, the culture medium was changed from that for hNCC to αMEM with 10% FBS (Figure 7A), which we used previously for human bone marrow-derived MSCs (hBM-MSCs) [24]. Through the induction of hMSCs, the expression of *NGFR* and *SOX10* reduced rapidly within 48 hours (PN0) of the medium change, while that of *PAX3* and *TFAP2A* reduced gradually until passage 3 (Figure S5A). Conversely, the expression of MSC markers (*CD73*, *CD105*, and *CD44*) increased rapidly within 48 hours, reached a maximum by passage number 2, and maintained their expression at a level comparable to that in BMMSCs (Figure S5B). These results indicated that the transition from NCCs to MSCs was gradual during passage number three. Cells passaged three times in the medium showed a typical fibroblastic morphology similar to that of hMSCs (Figure 7B), and expressed surface markers for hMSCs (positive for CD73, CD105, and CD44, and negative for CD45) (Figure 7C). Microarray analyses revealed that hNCC-derived MSCs had a global expression pattern similar to that of primary hBM-MSCs (Figure 7D). Differentiation properties toward osteogenic, chondrogenic, and adipogenic lineages are one of the criteria required for MSCs [42], which were clearly confirmed in hNCC-derived MSCs (Figure 7E). FACS analysis showed that there was no SSEA4-positive cells (Figure 7F) and the expression of PSC marker genes was below detectable levels (Figure 7G).

### Derivation of osteogenic cells from hiPSCs under defined culture conditions

**Figure 8:**
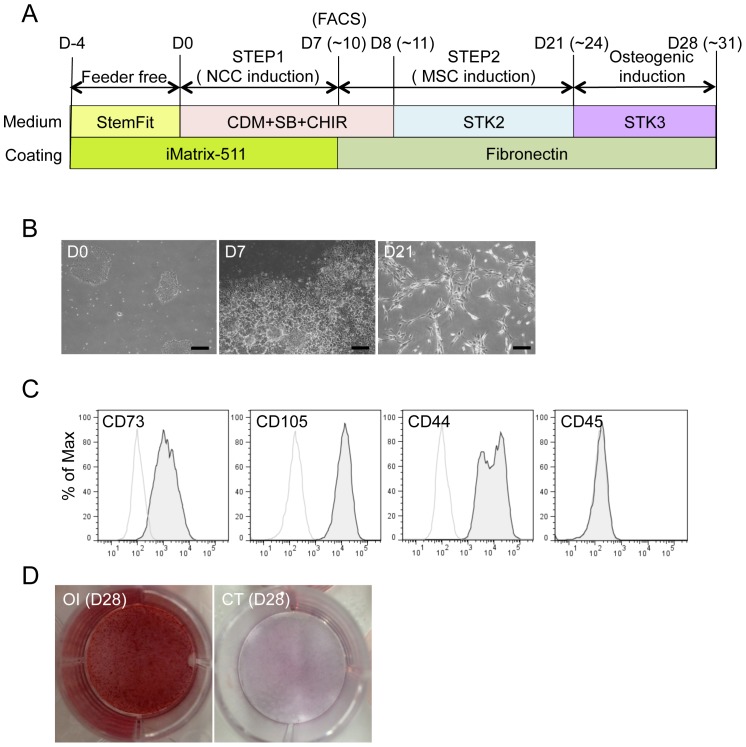
Derivation of hMSCs from hNCCs under defined culture conditions. A) Schematic protocol for the induction of hMSCs from hNCCs under defined culture conditions. B) Phase contrast images of cells 0, 7, and 21 days after the hNCC and hMSC induction, respectively. Scale bar, 200 µm. C) Expression of hMSC-related surface markers in hMSCs induced under defined culture conditions. D) Osteogenic differentiation (OI) properties of hMSCs induced under defined culture conditions. hMSCs were cultured during the induction period in STK2 as a control.

We determined the feasibility of inducing terminally differentiated cells from iPSCs under defined culture conditions (Figure 8). 987A3 hiPSCs were used as the initial material, which have been generated and maintained under feeder-free and xeno-free conditions [21]. Cells were dissociated into single cells, seeded on iMatrix-coated dishes (0.83–1.35 cells/cm^2^), and cultured with StemFit medium for five days. hNCCs were then induced for seven to ten days (Figure 8A). The efficiency of hNCC induction under these conditions was 40.9±5.5% (± SD. N = 3, biological triplicate). The induction of hMSCs was performed using CDM for MSCs (STK2) instead of αMEM/10% FBS (Figure 8A). After several passages of hNCCs in STK2, the morphology of cells changed from cuboidal to fibroblastic, similar to that of hBM-MSCs (Figure 8B). The expression patterns of surface markers were compatible with those of hMSCs (positive for CD73, CD105, and CD44, and negative for CD45) (Figure 8C) and the differentiation properties for osteogenic, chondrogenic, and adipogenic lineages were confirmed when the standard FBS-containing induction medium was used (Figure S6). Osteogenic differentiation was also confirmed using the chemically-defined osteogenic medium (STK3) (Figure 8D). These results indicated that all steps from iPSC to osteogenic cells could be performed under defined culture conditions.

## Discussion

In the present study, we developed a simple and efficient induction method for hNCCs from hPSCs. The induction efficiency of this method was high (70–80%) irrespective with the type of hPSC. The induced hNCCs exhibited the cranial NCC characters under maintenance culture conditions, while further treatment with insulin and RA marginally posteriorized hNCCs. Consistent with the expression of cranial NCC markers, induced hNCCs could differentiate into corneal endothelial cells, which is a characteristic of cranial NCCs.

Our protocol was independent of the BMP signal. In our protocol, DMH1, a specific BMP inhibitor, clearly attenuated the induction efficiency of the p75^high^ fraction (Figure S1). This result clearly contradicted the findings of previous studies (no effect [14] or increased efficiency [15]). The marked differences in the findings of these studies may be attributed to the seeding density used at the beginning of induction. The seeding density of our protocol was approximately 2–4 clumps/cm^2^ (approximately 20 cells/cm^2^), while other studies used 1×10^4^ cells/cm^2^ [26]. Both CNS and neural crest fates were previously observed when cells were seeded at a low density, while CNS cells primarily formed at a high density [43]. In accordance with these findings, the efficiency of the NCC induction was markedly decreased if clumps were seeded at a higher density (data not shown). The high density of hNCCs may have exaggerated local BMP signaling secreted from the hNCCs themselves. Therefore, we combined high density seeding with the BMP inhibitor treatment; however, the efficiency was still low (data not shown). Based on these results, we could not account for the differences between our protocol and those of previous studies.

In order to compare the hNCCs in this study with those in previous studies, we analyzed gene expression profiles of hNCCs published previously. The comparison of the relative induction levels of NCC specific genes revealed that hNCCs differentiated by our protocol and previous studies showed similarities in some aspects, but overall profiles were different from each other (Figure S3). These results indicated that induction protocols reported in this study and in the previous studies induced different subset of hNCCs.

Induced hNCCs exhibited differentiation properties for multiple cell lineages including peripheral neurons, glial cells, melanocytes, and corneal endothelial cells, and also delivered hMSCs that further differentiated into osteogenic, chondrogenic, and adipogenic cells. These properties are compatible with NCCs being multipotent stem cells [3]. However, clonal analyses are indispensable for confirming the stemness of induced hNCCs. Previous clonal analyses revealed that 63–65% of the hNCC clones could differentiated into multi-lineage cells positive for markers of neurons, glial cells, and smooth muscle cells [43], [44], suggesting that hNCCs induced from hPSCs were multipotent on the clonal level. Although stemness has yet to be investigated in this study, induced hNCCs in this protocol will be a promising cell source for various types of research.

Human diseases that have been related to the development of hNCCs include Hirschsprung's disease, DiGeorge syndrome, Waardenburg syndrome, Charcot-Marie-tooth disease, Hermansky-Pudlak syndrome, familial dysautonomia, Chediak-Higashi syndrome, and CHARGE syndrome [45], [46]. hNCCs containing the mutations responsible for these diseases can be induced from hiPSCs established from the respective patients; therefore, this will be a powerful tool for creating *in vitro* disease models that can contribute to a more detailed understanding of the pathogenesis of NCC disorders and also to the development of novel therapeutic modalities [15]. In addition, hNCCs have been shown to be the cell-of-origin of some cancers such as neuroblastoma [47], which indicates that hNCCs can be used in *in vitro* transformation experiments. We have already confirmed that the survival rate of freeze-stocked hNCCs was satisfactory and the freeze and thaw process had no impact on the growth and differentiation properties of these cells (data not shown). These are favorable features for a material in research because it is important to use cells of the same quality in order to evaluate reproducibility.

Induced hNCCs-deridatives can also be used for cell therapy. In this regard, hNCC-derived hMSCs will be a very useful material. hMSCs have been used in a wide range of regenerative medicines, and promising results have been reported in some cases [48], [49]. In contrast with the advances reported in clinical applications, many issues related to the biology of hMSCs have yet to be investigated, one of which is the cell-of-origin of hMSCs. hNCCs may be the precursors of hMSCs based on the finding that craniofacial skeletal tissues are derived from NCCs [50]. This has also been supported in lineage tracing experiments using P0-cre mice [51], [52]. Current sources of hMSCs include bone marrow, fat tissue, synovium, and umbilical cord; however, it remains unclear whether NCC-derived cells exist in all of these adult tissues and serve as the source of hMSCs. A comparison between hNCC-derived MSCs and somatic tissue-derived hMSCs may provide more information related to this issue.

One of the limitations of current hMSCs is their limited proliferative activity, which may pose problems in their application to conditions requiring a large amount of cells. This can be overcome if hNCC-derived MSCs are used because hNCCs can be induced from hPSCs, which have unlimited proliferative activity. Two issues are important for this application. One is to be free from infectious substances that may be derived from animal materials. Using iPSCs generated and maintained under feeder-free and xeno-free conditions, we successfully induced hNCCs and hMSCs with minimum animal material (BSA in CDM) (Figure 8A). Furthermore, we generated terminally differentiated cells (osteogenic cells) from these MSCs under chemically defined media. To the best of our knowledge, this is the first study to demonstrate the induction of osteogenic cells under feeder-free and serum-free conditions from PSCs. The other concern relates to the contamination of undifferentiated cells, particularly parental hPSCs, which may lead to serious conditions such as the formation of malignant tumors [53]. We confirmed that hNCC-derived hMSCs were free from SSEA4-expressing cells and the expression of PSC-marker genes was below detectable levels (Figures 7F, G). Although more precise and meticulous analyses are required to prove the safety of these cells, the results of the present study have provided evidence to promote the use of hNCC-derived hMSCs for cell therapy.

## Supporting Information

Figure S1 **Effect of the BMP signal on the induction of p75^high^ cells.** hiPSCs (201B7) were treated in NCC induction media with BMP4 (10 ng/ml) (A) or DMH1 (10 µM) (B), and the fraction of p75-positive cells was analyzed by FACS. C) Effects of BMP signal inhibitors on the induction of p75^high^ cells. 201B7 cells were treated with each BMP inhibitor at the indicated dosage, and the fraction of p75-positive cells was analyzed by FACS.
(TIFF)

Figure S2 **Global comparison of the expressions of genes between PSCs and p75^high^ cells.** A) A volcano plot showing the P value for differences in the expression of each gene between the average of PSC lines (H9, KhES1, 414C2, and 201B7) and the average of corresponding p75^high^ cells. A total of 562 entities downregulated and 447 entities upregulated in p75^high^ cells were identified as a differentially expressed gene set. B) Heat map analyses revealed global similarities among hNCCs derived from each PSC line.
(TIFF)

Figure S3 **Expression of NCC marker genes in induced NCCs from PSCs.** The induction ratio of NCC markers relative to a corresponding pluripotent baseline was demonstrated in each induced NCC. iPS NCCs, GSE44727. WA09_NC_Day11, 45223. Marker genes for each sub-population of NCC were labeled using the indicated colors.
(TIFF)

Figure S4 **Comparison of gene expression profiles between hNCCs at different passages by scatter plotting.** RNAs were extracted from hNCCs derived from 201B7 (A) and KhES1 (B) at different passages (PN0, PN4 and PN10), and analyzed using microarrays. C) Correlation coefficient analysis was performed using these data.
(TIFF)

Figure S5 **The expression of markers for hNCCs and hMSCs in each passage.** A gradual transition from hNCCs to hMSCs was observed in hNCC markers (A) and hMSC markers (B). Average ± SD. N = 3, biological triplicates. Regarding BMSCs, cDNA was prepared from the bone marrow stromal cells of four healthy donors (BM25, 26, 34, and 107), and the average was presented as BMSCs in each graph.
(TIFF)

Figure S6 **Osteogenic-, chondrogenic-, adipogenic induction from feeder-free hiPSCs through hNCC-derived hMSCs.** Differentiation properties of hNCC-MSCs. The induction for osteogenic (OI), chondrogenic (CI), and adipogenic (AI) lineages was performed as described in the Materials and Methods section and evaluated by Alizarin Red staining (OI), Alcian Blue staining (CI), and Oil Red O staining (AI), respectively. Scale bar, 200 µm.
(TIFF)

Table S1 Information of primary antibodies used in this study.
(TIF)

Table S2 Information of PCR primers used in this study.
(TIF)

## References

[1] Liu Z, Tang Y, Lu S, Zhou J, Du Z, et al (2013) The tumourigenicity of iPS cells and their differentiated derivates. J Cell Mol Med 17:782–791. 23711115 10.1111/jcmm.12062 PMC3823182

[2] Le Douarin NM, Dupin E (2003) Multipotentiality of the neural crest. Curr Opin Genet Dev 13:529–536. 14550420 10.1016/j.gde.2003.08.002

[3] Sauka-Spengler T, Bronner-Fraser M (2008) A gene regulatory network orchestrates neural crest formation. Nat Rev Mol Cell Biol 9:557–568. 18523435 10.1038/nrm2428

[4] Kalcheim C, Burstyn-Cohen T (2005) Early stages of neural crest ontogeny: formation and regulation of cell delamination. Int J Dev Biol 49:105–116. 15906222 10.1387/ijdb.041949ck

[5] Kalcheim C (2000) Mechanisms of early neural crest development: from cell specification to migration. Int Rev Cytol 200:143–196. 10965468 10.1016/s0074-7696(00)00004-8

[6] Vincent SD, Buckingham ME (2010) How to make a heart: the origin and regulation of cardiac progenitor cells. Curr Top Dev Biol 90:1–41. 20691846 10.1016/S0070-2153(10)90001-X

[7] Neirinckx V, Coste C, Rogister B, Wislet-Gendebien S (2013) Concise review: adult mesenchymal stem cells, adult neural crest stem cells, and therapy of neurological pathologies: a state of play. Stem Cells Transl Med 2:284–296. 23486833 10.5966/sctm.2012-0147 PMC3659839

[8] Neirinckx V, Marquet A, Coste C, Rogister B, Wislet-Gendebien S (2013) Adult bone marrow neural crest stem cells and mesenchymal stem cells are not able to replace lost neurons in acute MPTP-lesioned mice. PLoS One 8:e64723. 23741377 10.1371/journal.pone.0064723 PMC3669410

[9] Giuliani M, Oudrhiri N, Noman ZM, Vernochet A, Chouaib S, et al (2011) Human mesenchymal stem cells derived from induced pluripotent stem cells down-regulate NK-cell cytolytic machinery. Blood 118:3254–3262. 21803852 10.1182/blood-2010-12-325324

[10] Villa-Diaz LG, Brown SE, Liu Y, Ross AM, Lahann J, et al (2012) Derivation of mesenchymal stem cells from human induced pluripotent stem cells cultured on synthetic substrates. Stem Cells 30:1174–1181. 22415987 10.1002/stem.1084 PMC3549569

[11] Liu Q, Spusta SC, Mi R, Lassiter RN, Stark MR, et al (2012) Human neural crest stem cells derived from human ESCs and induced pluripotent stem cells: induction, maintenance, and differentiation into functional schwann cells. Stem Cells Transl Med 1:266–278. 23197806 10.5966/sctm.2011-0042 PMC3659695

[12] Chimge NO, Bayarsaihan D (2010) Generation of neural crest progenitors from human embryonic stem cells. J Exp Zool B Mol Dev Evol 314:95–103. 19780036 10.1002/jez.b.21321

[13] Milet C, Monsoro-Burq AH (2012) Embryonic stem cell strategies to explore neural crest development in human embryos. Dev Biol 366:96–99. 22306197 10.1016/j.ydbio.2012.01.016

[14] Menendez L, Yatskievych TA, Antin PB, Dalton S (2011) Wnt signaling and a Smad pathway blockade direct the differentiation of human pluripotent stem cells to multipotent neural crest cells. Proc Natl Acad Sci U S A 108:19240–19245. 22084120 10.1073/pnas.1113746108 PMC3228464

[15] Mica Y, Lee G, Chambers SM, Tomishima MJ, Studer L (2013) Modeling neural crest induction, melanocyte specification, and disease-related pigmentation defects in hESCs and patient-specific iPSCs. Cell Rep 3:1140–1152. 23583175 10.1016/j.celrep.2013.03.025 PMC3681528

[16] Suemori H, Yasuchika K, Hasegawa K, Fujioka T, Tsuneyoshi N, et al (2006) Efficient establishment of human embryonic stem cell lines and long-term maintenance with stable karyotype by enzymatic bulk passage. Biochem Biophys Res Commun 345:926–932. 16707099 10.1016/j.bbrc.2006.04.135

[17] Amit M, Carpenter MK, Inokuma MS, Chiu CP, Harris CP, et al (2000) Clonally derived human embryonic stem cell lines maintain pluripotency and proliferative potential for prolonged periods of culture. Dev Biol 227:271–278. 11071754 10.1006/dbio.2000.9912

[18] Okita K, Matsumura Y, Sato Y, Okada A, Morizane A, et al (2011) A more efficient method to generate integration-free human iPS cells. Nat Methods 8:409–412. 21460823 10.1038/nmeth.1591

[19] Takahashi K, Tanabe K, Ohnuki M, Narita M, Ichisaka T, et al (2007) Induction of pluripotent stem cells from adult human fibroblasts by defined factors. Cell 131:861–872. 18035408 10.1016/j.cell.2007.11.019

[20] McMahon AP, Bradley A (1990) The Wnt-1 (int-1) proto-oncogene is required for development of a large region of the mouse brain. Cell 62:1073–1085. 2205396 10.1016/0092-8674(90)90385-r

[21] Nakagawa M, Taniguchi Y, Senda S, Takizawa N, Ichisaka T, et al (2014) A novel efficient feeder-free culture system for the derivation of human induced pluripotent stem cells. Sci Rep 4:3594. 24399248 10.1038/srep03594 PMC3884228

[22] Nasu A, Ikeya M, Yamamoto T, Watanabe A, Jin Y, et al (2013) Genetically matched human iPS cells reveal that propensity for cartilage and bone differentiation differs with clones, not cell type of origin. PLoS One 8:e53771. 23382851 10.1371/journal.pone.0053771 PMC3561398

[23] Wataya T, Ando S, Muguruma K, Ikeda H, Watanabe K, et al (2008) Minimization of exogenous signals in ES cell culture induces rostral hypothalamic differentiation. Proc Natl Acad Sci U S A 105:11796–11801. 18697938 10.1073/pnas.0803078105 PMC2575295

[24] Colleoni S, Galli C, Giannelli SG, Armentero MT, Blandini F, et al (2010) Long-term culture and differentiation of CNS precursors derived from anterior human neural rosettes following exposure to ventralizing factors. Exp Cell Res 316:1148–1158. 20171210 10.1016/j.yexcr.2010.02.013

[25] James MJ, Jarvinen E, Wang XP, Thesleff I (2006) Different roles of Runx2 during early neural crest-derived bone and tooth development. J Bone Miner Res 21:1034–1044. 16813524 10.1359/jbmr.060413

[26] Lee G, Chambers SM, Tomishima MJ, Studer L (2010) Derivation of neural crest cells from human pluripotent stem cells. Nat Protoc 5:688–701. 20360764 10.1038/nprot.2010.35

[27] Ohta S, Imaizumi Y, Okada Y, Akamatsu W, Kuwahara R, et al (2011) Generation of human melanocytes from induced pluripotent stem cells. PLoS One 6:e16182. 21249204 10.1371/journal.pone.0016182 PMC3020956

[28] Fang D, Leishear K, Nguyen TK, Finko R, Cai K, et al (2006) Defining the conditions for the generation of melanocytes from human embryonic stem cells. Stem Cells 24:1668–1677. 16574754 10.1634/stemcells.2005-0414

[29] Ju C, Zhang K, Wu X (2012) Derivation of corneal endothelial cell-like cells from rat neural crest cells in vitro. PLoS One 7:e42378. 22860120 10.1371/journal.pone.0042378 PMC3409168

[30] Umeda K, Zhao J, Simmons P, Stanley E, Elefanty A, et al (2012) Human chondrogenic paraxial mesoderm, directed specification and prospective isolation from pluripotent stem cells. Sci Rep 2:455. 22701159 10.1038/srep00455 PMC3374161

[31] Okamoto T, Aoyama T, Nakayama T, Nakamata T, Hosaka T, et al (2002) Clonal heterogeneity in differentiation potential of immortalized human mesenchymal stem cells. Biochem Biophys Res Commun 295:354–361. 12150956 10.1016/s0006-291x(02)00661-7

[32] Kreitzer FR, Salomonis N, Sheehan A, Huang M, Park JS, et al (2013) A robust method to derive functional neural crest cells from human pluripotent stem cells. Am J Stem Cells 2:119–131. 23862100 PMC3708511

[33] Lee G, Ramirez CN, Kim H, Zeltner N, Liu B, et al (2012) Large-scale screening using familial dysautonomia induced pluripotent stem cells identifies compounds that rescue IKBKAP expression. Nat Biotechnol 30:1244–1248. 23159879 10.1038/nbt.2435 PMC3711177

[34] Kimura C, Takeda N, Suzuki M, Oshimura M, Aizawa S, et al (1997) Cis-acting elements conserved between mouse and pufferfish Otx2 genes govern the expression in mesencephalic neural crest cells. Development 124:3929–3941. 9374391 10.1242/dev.124.20.3929

[35] Qiu M, Bulfone A, Ghattas I, Meneses JJ, Christensen L, et al (1997) Role of the Dlx homeobox genes in proximodistal patterning of the branchial arches: mutations of Dlx-1, Dlx-2, and Dlx-1 and -2 alter morphogenesis of proximal skeletal and soft tissue structures derived from the first and second arches. Dev Biol 185:165–184. 9187081 10.1006/dbio.1997.8556

[36] Manley NR, Capecchi MR (1995) The role of Hoxa-3 in mouse thymus and thyroid development. Development 121:1989–2003. 7635047 10.1242/dev.121.7.1989

[37] Liu Z, Yu S, Manley NR (2007) Gcm2 is required for the differentiation and survival of parathyroid precursor cells in the parathyroid/thymus primordia. Dev Biol 305:333–346. 17382312 10.1016/j.ydbio.2007.02.014 PMC1931567

[38] Motohashi T, Aoki H, Yoshimura N, Kunisada T (2006) Induction of melanocytes from embryonic stem cells and their therapeutic potential. Pigment Cell Res 19:284–289. 16827747 10.1111/j.1600-0749.2006.00317.x

[39] Johnston MC, Noden DM, Hazelton RD, Coulombre JL, Coulombre AJ (1979) Origins of avian ocular and periocular tissues. Exp Eye Res 29:27–43. 510425 10.1016/0014-4835(79)90164-7

[40] Trainor PA, Tam PP (1995) Cranial paraxial mesoderm and neural crest cells of the mouse embryo: co-distribution in the craniofacial mesenchyme but distinct segregation in branchial arches. Development 121:2569–2582. 7671820 10.1242/dev.121.8.2569

[41] Fitch JM, Birk DE, Linsenmayer C, Linsenmayer TF (1990) The spatial organization of Descemet's membrane-associated type IV collagen in the avian cornea. J Cell Biol 110:1457–1468. 2182654 10.1083/jcb.110.4.1457 PMC2116101

[42] De Schauwer C, Meyer E, Van de Walle GR, Van Soom A (2011) Markers of stemness in equine mesenchymal stem cells: a plea for uniformity. Theriogenology 75:1431–1443. 21196039 10.1016/j.theriogenology.2010.11.008

[43] Lee G, Kim H, Elkabetz Y, Al Shamy G, Panagiotakos G, et al (2007) Isolation and directed differentiation of neural crest stem cells derived from human embryonic stem cells. Nat Biotechnol 25:1468–1475. 18037878 10.1038/nbt1365

[44] Curchoe CL, Maurer J, McKeown SJ, Cattarossi G, Cimadamore F, et al (2010) Early acquisition of neural crest competence during hESCs neuralization. PLoS One 5:e13890. 21085480 10.1371/journal.pone.0013890 PMC2976694

[45] Bajpai R, Chen DA, Rada-Iglesias A, Zhang J, Xiong Y, et al (2010) CHD7 cooperates with PBAF to control multipotent neural crest formation. Nature 463:958–962. 20130577 10.1038/nature08733 PMC2890258

[46] Lee G, Papapetrou EP, Kim H, Chambers SM, Tomishima MJ, et al (2009) Modelling pathogenesis and treatment of familial dysautonomia using patient-specific iPSCs. Nature 461:402–406. 19693009 10.1038/nature08320 PMC2784695

[47] Jiang M, Stanke J, Lahti JM (2011) The connections between neural crest development and neuroblastoma. Curr Top Dev Biol 94:77–127. 21295685 10.1016/B978-0-12-380916-2.00004-8 PMC3633592

[48] Caplan AI (2007) Adult mesenchymal stem cells for tissue engineering versus regenerative medicine. J Cell Physiol 213:341–347. 17620285 10.1002/jcp.21200

[49] Silva NA, Sousa N, Reis RL, Salgado AJ (2013) From basics to clinical: A comprehensive review on spinal cord injury. Prog Neurobiol. 10.1016/j.pneurobio.2013.11.002 24269804

[50] Helms JA, Schneider RA (2003) Cranial skeletal biology. Nature 423:326–331. 12748650 10.1038/nature01656

[51] Morikawa S, Mabuchi Y, Niibe K, Suzuki S, Nagoshi N, et al (2009) Development of mesenchymal stem cells partially originate from the neural crest. Biochem Biophys Res Commun 379:1114–1119. 19161980 10.1016/j.bbrc.2009.01.031

[52] Takashima Y, Era T, Nakao K, Kondo S, Kasuga M, et al (2007) Neuroepithelial cells supply an initial transient wave of MSC differentiation. Cell 129:1377–1388. 17604725 10.1016/j.cell.2007.04.028

[53] Cai J, Yang M, Poremsky E, Kidd S, Schneider JS, et al (2010) Dopaminergic neurons derived from human induced pluripotent stem cells survive and integrate into 6-OHDA-lesioned rats. Stem Cells Dev 19:1017–1023. 19824823 10.1089/scd.2009.0319 PMC3135248

