# Supplementary figures and images for: Derivation of Mesenchymal Stromal Cells from Pluripotent Stem Cells through a Neural Crest Lineage using Small Molecule Compounds with Defined Media
Source: PLoS One. 2014 Dec 2;9(12):e112291. doi: 10.1371/journal.pone.0112291 (PMC4251837; doi:10.1371/journal.pone.0112291)

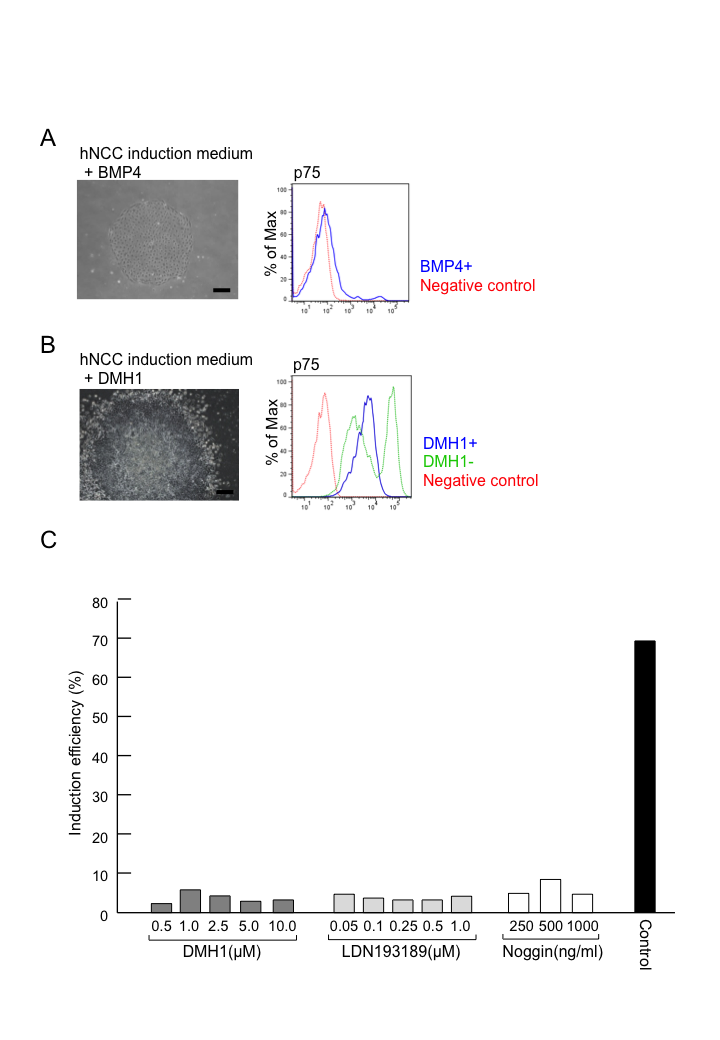

Supplement: Figure S1 — Effect of the BMP signal on the induction of p75high cells. hiPSCs (201B7) were treated in NCC induction media with BMP4 (10 ng/ml) (A) or DMH1 (10 µM) (B), and the fraction of p75-positive cells was analyzed by FACS. C) Effects of BMP signal inhibitors on the induction of p75high cells. 201B7 cells were treated with each BMP inhibitor at the indicated dosage, and the fraction of p75-positive cells was analyzed by FACS. (TIFF) [file pone.0112291.s001.tiff]

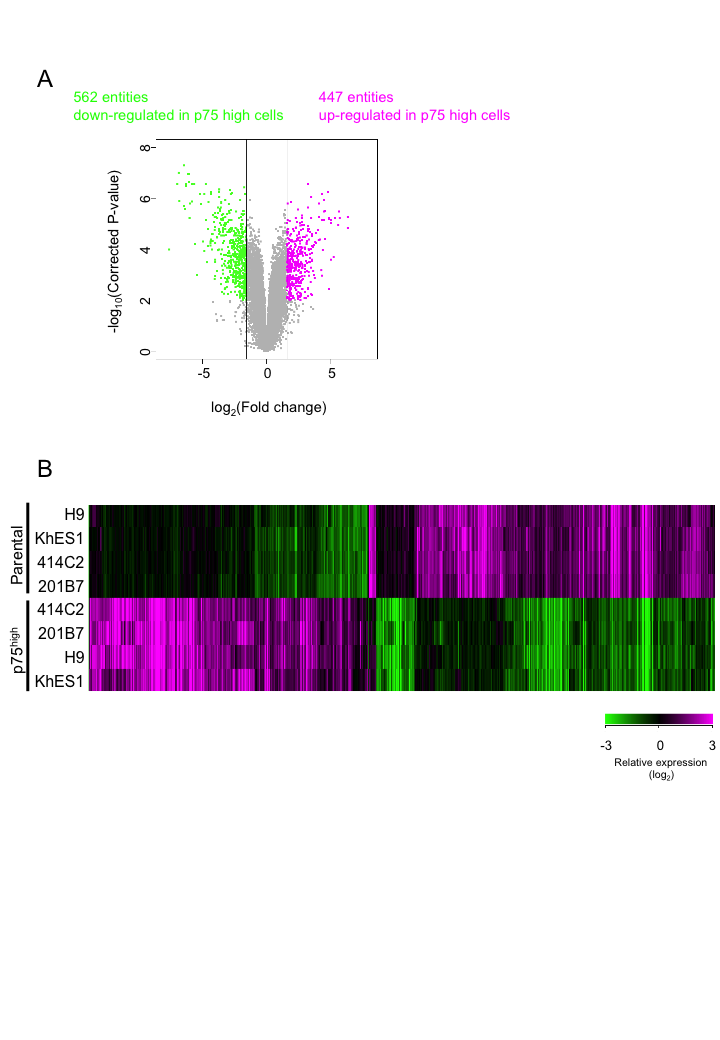

Supplement: Figure S2 — Global comparison of the expressions of genes between PSCs and p75high cells. A) A volcano plot showing the P value for differences in the expression of each gene between the average of PSC lines (H9, KhES1, 414C2, and 201B7) and the average of corresponding p75high cells. A total of 562 entities downregulated and 447 entities upregulated in p75high cells were identified as a differentially expressed gene set. B) Heat map analyses revealed global similarities among hNCCs derived from each PSC line. (TIFF) [file pone.0112291.s002.tiff]

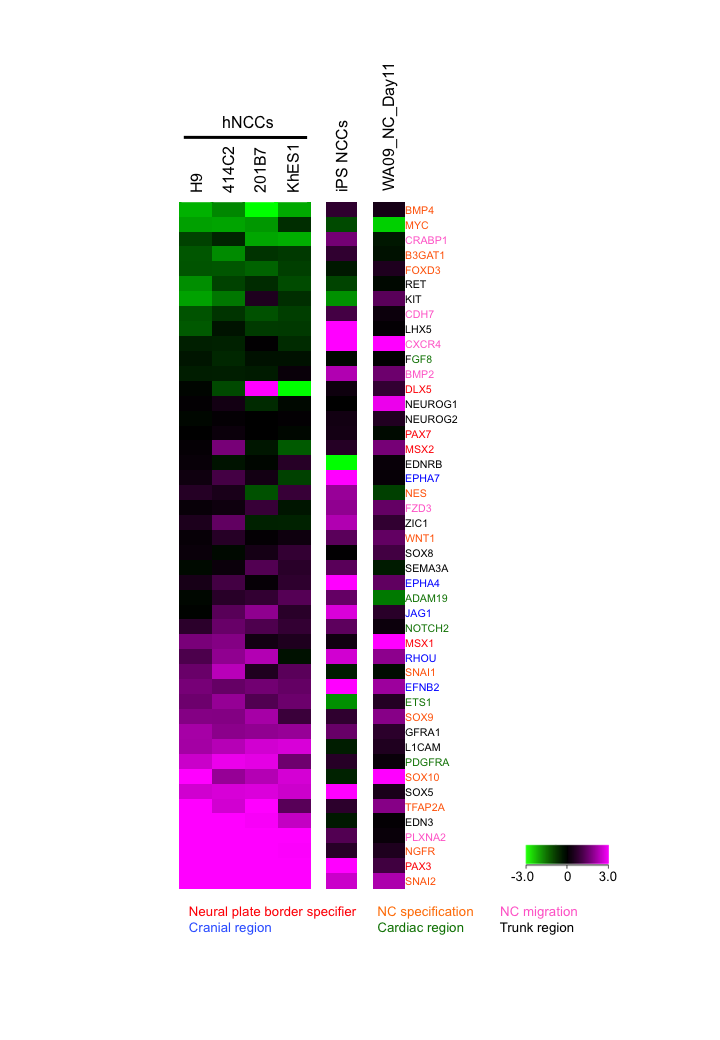

Supplement: Figure S3 — Expression of NCC marker genes in induced NCCs from PSCs. The induction ratio of NCC markers relative to a corresponding pluripotent baseline was demonstrated in each induced NCC. iPS NCCs, GSE44727. WA09_NC_Day11, 45223. Marker genes for each sub-population of NCC were labeled using the indicated colors. (TIFF) [file pone.0112291.s003.tiff]

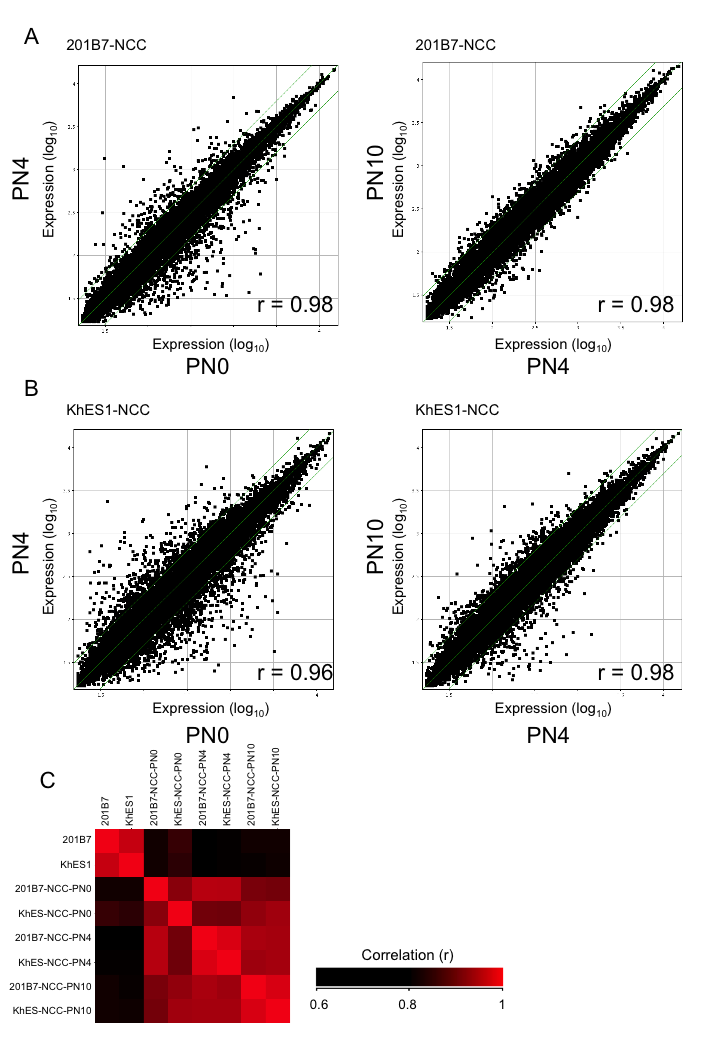

Supplement: Figure S4 — Comparison of gene expression profiles between hNCCs at different passages by scatter plotting. RNAs were extracted from hNCCs derived from 201B7 (A) and KhES1 (B) at different passages (PN0, PN4 and PN10), and analyzed using microarrays. C) Correlation coefficient analysis was performed using these data. (TIFF) [file pone.0112291.s004.tiff]

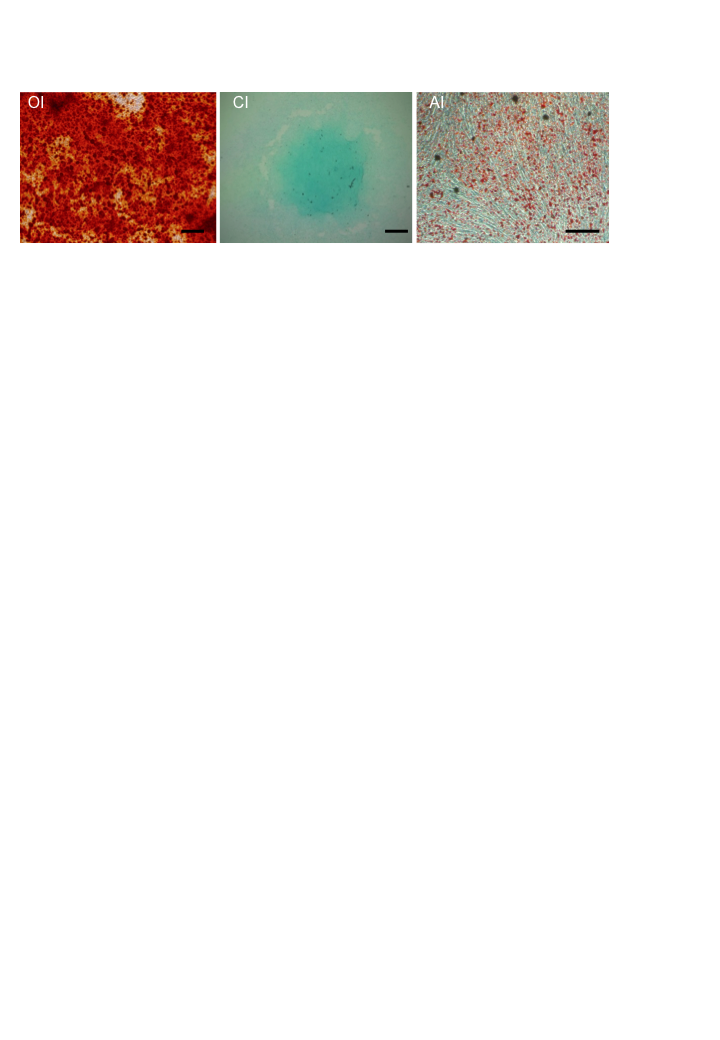

Supplement: Figure S6 — Osteogenic-, chondrogenic-, adipogenic induction from feeder-free hiPSCs through hNCC-derived hMSCs. Differentiation properties of hNCC-MSCs. The induction for osteogenic (OI), chondrogenic (CI), and adipogenic (AI) lineages was performed as described in the Materials and Methods section and evaluated by Alizarin Red staining (OI), Alcian Blue staining (CI), and Oil Red O staining (AI), respectively. Scale bar, 200 µm. (TIFF) [file pone.0112291.s006.tiff]

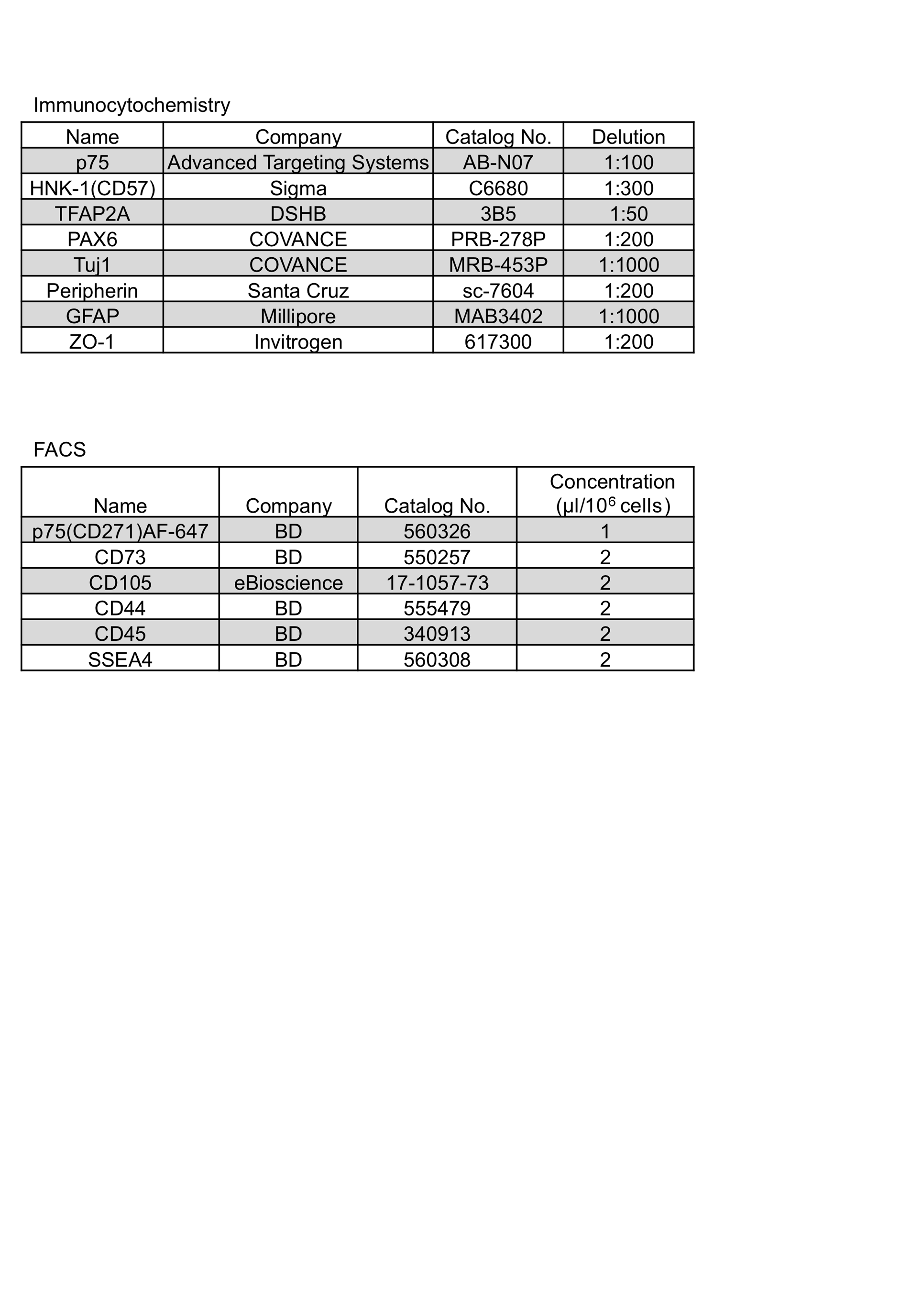

Supplement: Table S1 — Information of primary antibodies used in this study. (TIF) [file pone.0112291.s007.tif]

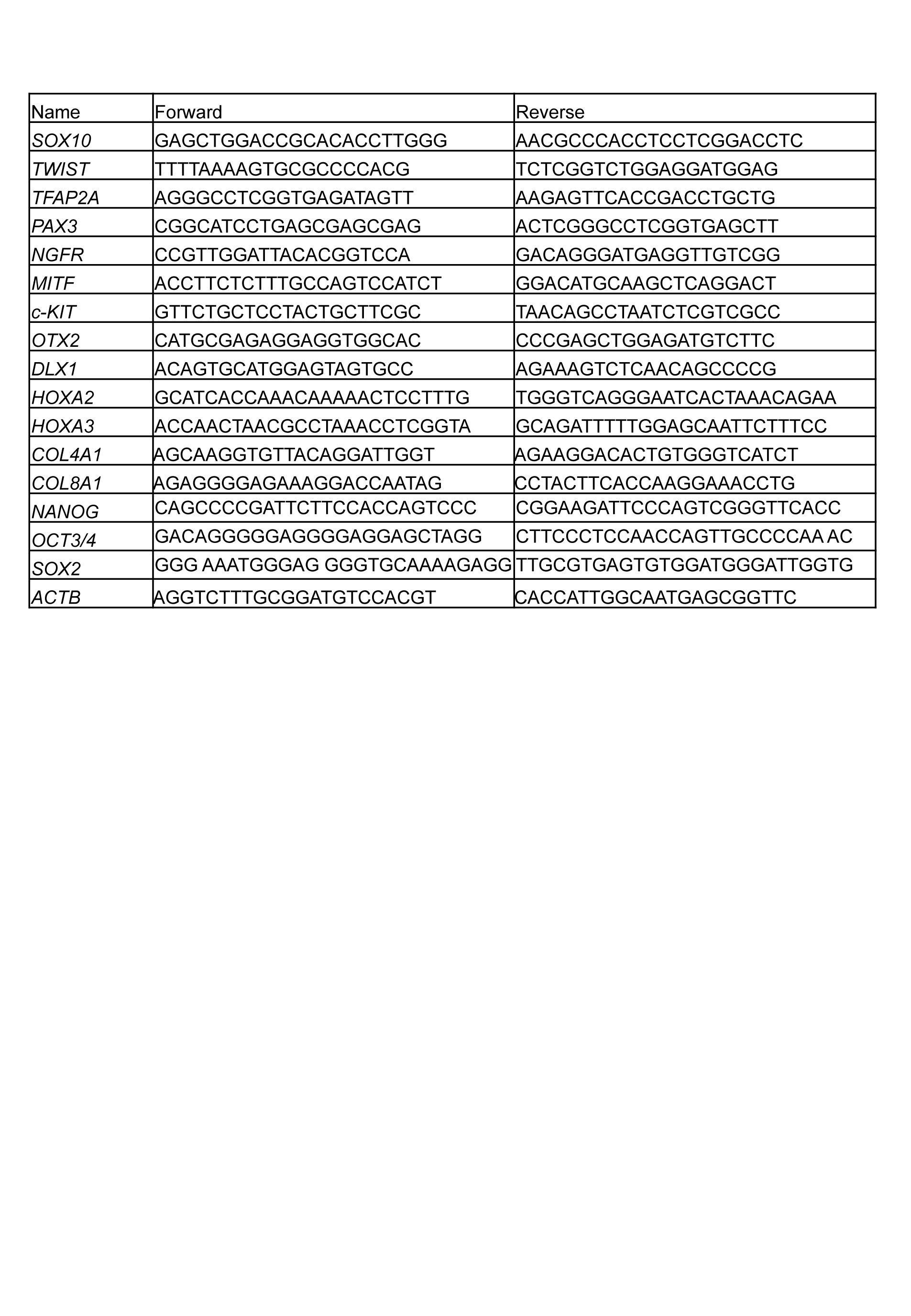

Supplement: Table S2 — Information of PCR primers used in this study. (TIF) [file pone.0112291.s008.tif]
